# Pediatric Asthma in the Inland Empire: Environmental Burden, Gaps in Preventive Care, and Unmet Needs

**DOI:** 10.3390/children12091183

**Published:** 2025-09-04

**Authors:** Catherine Kim, Christine Gharib, Hani Atamna

**Affiliations:** California University of Science and Medicine, Colton, CA 92324, USA; christine.gharib@md.cusm.edu (C.G.); hani.atamna@cusm.edu (H.A.)

**Keywords:** pediatric asthma, air pollution, academic achievement, health care utilization, socioeconomic status, ozone, particulate matter, pest allergens, emergency department utilization

## Abstract

Background: Asthma is the most prevalent chronic illness in children worldwide, contributing to significant morbidity, health care utilization, and economic burden. In the United States, approximately five million children are affected by asthma. This review explores the environmental contexts and lifestyle determinants of pediatric asthma, with a focus on the Inland Empire (IE) region of Southern California. The IE’s unique geographic landscape and importance as a major transportation hub highlights its critical role for understanding how both environmental and structural factors exacerbate asthma burden within the pediatric population. Variables such as household income, parental education levels, and lack of community-based asthma programs were explored. Despite significant burdens, the IE remains under-represented in asthma research, contributing to persistent disparity. Methods: A narrative literature review and regional data analysis were conducted via PubMed, Scopus, and Google Scholar (2000–2025), alongside data from the CDC, CDPH, and American Lung Association. Key words used included “pediatric asthma, Inland Empire, air pollution, asthma disparity, emergency department utilization, socioeconomic status.” Inclusion criteria were: (1) studies or reports focusing on pediatric asthma (ages 0–17), (2) articles addressing environmental, socioeconomic, or health care-related risk factors, and (3) research with either national, state-level, or IE-specific data. Exclusion criteria were: (1) articles not in English, adult-only asthma studies, and (3) publications without original data or reference to pediatric asthma burden, management, or outcomes. Titles and abstracts were screened for relevance, and full texts were reviewed when abstracts met inclusion criteria. A total of 61 studies, reports, and data sources met this criterion and were included into this review. Results: The IE—comprised of San Bernardino (SB) and Riverside Counties— is home to four of the top five most polluted cities in North America. Vehicle emissions and industrial waste are concentrated in the region due to limited air circulation from surrounding mountains that entrap pollutants. Pediatric asthma ED visit rates in SB and Riverside were 60.5% and 59.3%, compared to California’s average of 56.7%. Hospitalization rates for children aged 0–4 were also higher in SB (24.4%) compared to the state average (17.3%). The elevated rates among school-aged children underscore the crucial need for interventions aimed at improving air quality, enhancing asthma management, and increasing access to preventive health care. Conclusions: Pediatric asthma in the IE reflects heightened environmental risks, socioeconomic barriers, and gaps in health care access. Addressing these disparities requires targeted interventions, policies, and region-specific research to enhance long-term management strategies and outcomes for vulnerable pediatric populations.

## 1. Introduction

Globally, asthma is considered the most common chronic disease affecting children (ages 0–17), contributing to substantial morbidity and excessive health care utilization across diverse regions, with up to 13-fold differences between countries [1]. The Global Asthma Report in 2022 estimated that over 262 million individuals are affected by asthma worldwide, and over a third (38% or 100 million) of these individuals are under the age of 15 [2]. By the end of 2025, it is projected that 400 million individuals will be affected by asthma, largely due to extrapolations from demographic shifts and environmental exposures [3]. The prevalence of asthma varies by region, with higher rates seen in high-income countries, and increasing trends can be witnessed in lower income regions due to rapid urbanization and environmental developments. Pediatric asthma not only imposes significant clinical and economic burdens, but also reflects broader inequities in diagnosis and treatment.

Pediatric asthma remains a pervasive and significant public health concern in the United States, with clinical, social, and economic implications. Nationally, asthma is reported to affect approximately five million children under the age of 18 [4]. In California, pediatric asthma prevalence has been studied, with a particular focus on urban centers such as Los Angeles [5]. The Inland Empire (IE), encompassing the Riverside and San Bernardino Counties of California, contains high levels of air pollution, elevated poverty rates, and relative shortages of health care resources, which have been identified as determinants of adverse asthma outcomes [6]. Pediatric asthma is one of the leading causes of chronic illness, emergency department (ED) admission, school absenteeism, and reduced quality of life [7]. Children with asthma will also have significantly more acute care visits and hospitalizations compared to non-asthmatic children, creating an economic burden. In 2022, pediatric asthma hospitalizations in the U.S. totaled USD 480.6 million in costs, with a mean cost of USD 7464 per hospitalization [8]. Over 40% of children with asthma will experience at least one exacerbation per year, and, on average, miss nine more school days per year compared to their peers without asthma. Beyond absenteeism, asthma can impair concentration, sleep quality, and physical participation in school activities, which are critical to learning and social development [9]. Uncontrolled asthma has been linked to lower standardized test scores, reduced classroom engagement, and increased likelihood of grade retention, which is a precursor for reduced academic performance [10,11]. Asthma is known to disproportionately affect low-income and racially segregated communities. Not only do disadvantaged groups experience extended burdens of air pollution, but they also have barriers to primary care and preventable services, leading to poorer asthma outcomes [12]. While numerous studies have examined pediatric asthma at the national and state levels, there is a striking lack of scholarship focused specifically on the IE. This review is distinct in that it synthesizes epidemiologic data, environmental exposures, and socioeconomic determinants unique to San Bernardino and Riverside Counties. By integrating both peer-reviewed literature and publicly available datasets, we highlight the IE as an under-represented region where high levels of air pollution intersect with poverty, housing quality, and limited health care infrastructure. We aim to examine the prevalence of asthma in the pediatric population of the San Bernardino and Riverside counties, with a particular focus on the status quo of care and gaps in management. This study seeks to underscore both structural and clinical barriers to effective asthma care within the IE, informing targeted interventions and public health initiatives to lessen the burden of asthma. To our knowledge, this is the first review to consolidate these diverse factors into a comprehensive regional perspective, thereby providing a framework for targeted interventions and future research priorities.

### 1.1. The Unmet Need: Pediatric Asthma Research in the Inland Empire Is Limited

Although progress has been made in understanding and managing pediatric asthma at both the national and local levels, there are many considerable disparities that persist in socioeconomically disadvantaged and environmentally burdened population groups. Children living closer to major railways, industrial sites, and public housing experience more poor air quality days and significantly more frequent asthma-related health care visits [13]. The intensified exposure of IE children to air pollution, coupled with challenging socioeconomic and systemic factors that limit their access to adequate health care, significantly exacerbates asthma disparities and adverse outcomes in the region. Children in under-represented regions of the IE who experience health insurance gaps also have 2.5-times higher odds of all-cause ED visits compared to continuously privately insured peers, due to being more likely to experience barriers in accessing consistent and high-quality health care, which may delay initial diagnosis and limit management options, hence, leading to worse outcomes [14]. However, despite these disparities and the environmental challenges to IE residents, there remains a conspicuous gap in research regarding pediatric asthma.

With regards to the gap in pediatric asthma research in the IE, it is crucial to note that despite an extensive search process, the most recent data—on both statewide and county levels—that was obtained dated back to 2022, per both the CDC and the California Health and Human Services Agency. There is a scarcity of up-to-date information on pediatric asthma in California as a whole, hindering the development of targeted interventions and delaying critical public health responses in a state with some of the highest asthma-related burdens in the country. With pediatric asthma rates disproportionately affecting communities in the IE, the lack of localized data collection and focused research on the IE impedes the ability to identify at-risk populations, allocate resources effectively, and implement timely, community-based prevention and education efforts. In a region already facing systemic health inequities, robust surveillance and reporting systems are crucial. Thus, this data gap is especially alarming in the IE.

### 1.2. In-Door and Out-Door Environmental Pollutants in the IE

Children in the IE are disproportionately exposed to air quality hazards, such as particulate matter (PM2.5) and ozone (Table 1) [6]. Thus, the under-representation of the IE in pediatric asthma research is concerning, given the region’s demographic composition and environmental strains. There also remains significant risk factors to indoor exposures, with children in high-pollution areas facing high exposure to in-door allergens as well—such as pest allergens, mold, and endotoxins—due to substandard housing conditions (Table 2) [15]. Increased barriers and burdens relating to increasing poverty and decreased housing quality further compound pediatric asthma risk. Region-specific epidemiologic data regarding pediatric asthma has not been extensively investigated and is contributing to the hindered development of tailored public health strategies and intervention programs for populations at risk.

By examining the current evidence on pediatric asthma at the national and state levels, we should be able to critically evaluate and define the specific contextual factors that affect the children residing in the IE. Our goal is to identify the urgent need for geographically targeted research initiatives to define public health intervention strategies for addressing the unique environmental and socioeconomic challenges faced by the underserved pediatric population of the IE.

## 2. National and Statewide Trends in Pediatric Asthma

Pediatric asthma continues to impact both quality of life and health care utilization in the pediatric population within the United States. According to the most recent data published by the Centers for Disease Control and Prevention (CDC), five million children (6.2% of the pediatric population) are living with asthma in 2022, with the highest prevalence remaining in the age group of 5–9 years old, estimated as affecting around 10–15% of this vulnerable population [16]. Despite minor declines in overall prevalence over the past decade, asthma remains the leading cause of emergency department (ED) visits, hospitalizations, missed school days, and health care costs in pediatric populations nationwide [17,18]. The American Lung Association (ALA) reports on the persistent burden of asthma, mentioning asthma-related ED visits remaining alarmingly high in pediatric populations [17,18].

Children diagnosed with asthma are at higher risk of exacerbations triggered by environmental factors, allergens, and respiratory and auditory infections, with 52.7% of children (2,383,631) experiencing at least one asthma attack in the last 12 months annually [16]. Examining the national trends reveals that improvements in the clinical management of asthma have not been evenly distributed across demographic groups, resulting in persistent disparity.

In California, asthma is a major chronic health concern in pediatric groups, even exceeding national patterns in some regions. According to the California Department of Public Health (CDPH), an estimated 12.4% of children have been diagnosed with asthma in 2022, which is higher than the national average of 6.2% and varies significantly based on geographical region [19]. In 2022, California pediatric asthma hospitalization was 8.9 per 10,000 children, while India and South Africa had rates ranging from 2.1–4.0 per 10,000 children, despite poorer air quality [20]. Concerningly, there are higher rates of asthma-related ED visits in certain parts of California [21]. Children living in the Central Valley, San Joaquin Valley, and the Inland Empire exhibit disproportionately higher rates of asthma morbidity up to 2.9-times greater compared to other regional and national averages. In 2010, asthma hospitalization rate for children was 9.7 per 10,000 children, which decreased to 8.9 per 10,000 children in 2022, showing an 8% decrease over 12 years [22]. Although data for hospitalization for pediatric asthma has shown a slow decline over the past decade, the rates are still substantially higher compared to countries with poorer air quality and higher rates of poverty. Environmental exposures to ozone and PM2.5 are believed to significantly contribute to these trends [23].

These patterns raise important questions regarding the disproportionately high prevalence of pediatric asthma cases in California, despite declining hospitalization rates and advancements in asthma management. One likely explanation lies in the intersection of persistent environmental exposures and systemic inequities. Regions such as the Central Valley and the IE are geographically predisposed to poor air dispersion, leading to higher concentrations of pollutants like ozone and PM2.5—both of which are well documented as significant asthma triggers. Compounding this, these regions house socioeconomically disadvantaged populations that may have limited access to consistent preventative care, live in substandard housing with increased indoor allergen exposure, and reside closer to major railway stations or industrial zones. These structural and environmental vulnerabilities in a state of stringent air quality regulations may explain why California’s pediatric asthma burden exceeds that of countries with objectively poorer air quality and higher poverty levels. Thus, it is important to consider that the observed trends likely reflect not only environmental exposures, but also deeper, more entrenched disparities in health care access, environmental justice, and social determinants of health.

**Figure 1:**
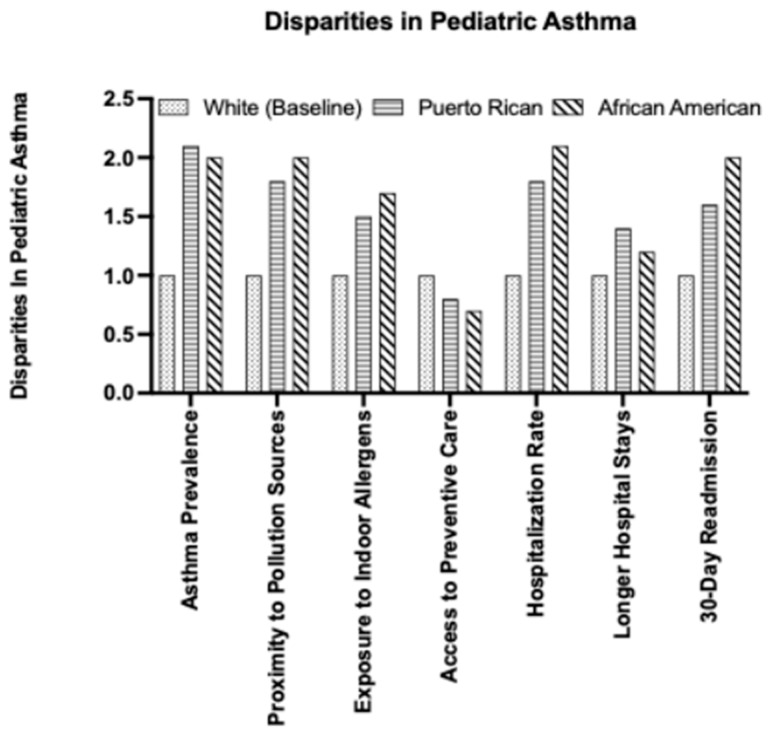
Population-dependent Variations in Pediatric Asthma Prevalence and Risk Factors within the United States. Prevalence and Disparities in pediatric asthma across several risk factors; Puerto Rican and African American children are at higher risk when compared to White population.

Pediatric asthma outcomes show disparities across racial, socioeconomic, and geographic lines both at the national and state levels [24,25,26,27]. African American and Puerto Rican children experience the highest rates of asthma prevalence, morbidity, and mortality (Figure 1). In California, African American children are more than twice as likely as White children to be hospitalized for asthma complications [28]. African American children with asthma are also 12% more likely to experience longer hospital stays and be readmitted within 30 days. Children in low-income households are more likely to live in conditions of sub-standard housing. These housing conditions have a higher indoor allergen exposure to mold, as well as closer proximity to highways and industrial facilities [24,25,26,27]. These risk factors derived from socioeconomic status are further compounded by limited access to consistent and high-quality health care, as well as established preventative services such as management visits and education programs.

Access to care remains a critical barrier to improving pediatric asthma outcomes. Children in underserved communities, particularly those without stable health insurance coverage, often face difficulties in accessing primary care providers, pulmonologists, and asthma specialists [29]. Underdiagnosis or delayed diagnosis of pediatric asthma is common in communities where health infrastructure is weak or health literacy is low [28]. These factors contribute to perpetuating a cycle of poorly controlled asthma and preventable complications. Thus, while there have been modest improvements in overall pediatric asthma management, significant disparities persist that require an investigative approach to identify causes and advance tailored interventions to address the unique needs of high-risk populations.

## 3. Regional Focus: The Inland Empire

The Inland Empire (IE), as we pointed out above, has consistently been listed as one of the top urban areas in the nation with the worst air pollution. Geographically, the IE is surrounded by various mountains, such as the San Bernardino mountains to the North, San Jacinto Mountains to the South, and Santa Ana Mountains to the West. Topographical features, in this case mountains, are known to trap pollutants and lead to ozone formation [30]. Hence, the IE is surrounded by mountains that entrap pollutants such as vehicle emissions and industrial waste, limiting air circulation. Per IQ Air’s 2024 World Air Quality Report, the top five most polluted regional cities in Northern America (i.e., defined as cities with a population ≥ 5000 in both the USA and Canada) were all located in California, with the cities consisting of Ontario with a PM2.5 (in µg/m^3^) of 14.3, Bloomington with 13.9, Huntington Park with 13.6, San Bernardino with 12.9, and Fontana with 12.7 (Table 3) [31].

Regarding Table 3, it is critical to underscore that four out of the five cities are located within the IE. For context, the current WHO global air quality guideline recommends an annual average PM2.5 concentration level of ≤5 µg/m^3^ [31]. Previous research has established an association between elevated PM2.5 levels and increased health risks, such as impairments in cardiovascular and respiratory functions [32]. Children residing in regions with a higher-than-average annual PM2.5 levels were more likely to have current asthma, experience acute asthma episodes, and/or have asthma-induced ED visits [33]. Air pollution is also known to stunt lung development, as indicated by clinically significant deficits in forced expiratory volume (FEV) [34]. Given the established association between poor lung function and mortality [35], it is imperative to identify the environmental, alongside the social and health care, barriers contributing to asthma exacerbations in the IE. Referring to the IE cities listed in Table 3, Fontana—the city with the lowest PM2.5 level in the list—had a level approximately 2.54-times greater than the recommended WHO guideline of ≤5 µg/m^3^, further underscoring the IE’s pervasive air quality and correspondingly elevated concern of exacerbated health effects.

**Figure 2:**
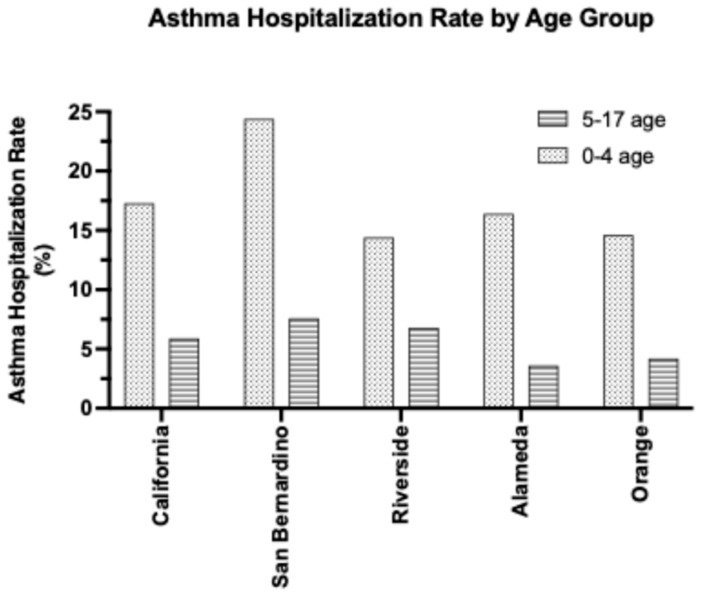
County–dependent variations in the rates of pediatric hospitalization (per 10,000 residents) due to asthma complications. Comparison of asthma hospitalization rates within the 0–4 age group vs. 5–17 age group shows increased trend in hospitalization in both groups and shows that children at age 0–4 are more likely to be hospitalized due to asthma complications in San Bernardino than any of the other counties (Source: California Breathing—the state asthma program run by California Dept. Of Health Care Access and Information).

In the status quo, the most recent pediatric asthma data is from 2022, highlighting a knowledge gap and the urgent need for updated research. As seen in Table 4 above, in 2022, the rate of asthma hospitalization among children aged 0–17 was 9.6% in San Bernardino County and 8.8% in Riverside County [22]. In comparison, other counties—such as Orange and Alameda—had hospitalization rates of 7.0% and 7.1%, respectively [22]. The above disparities highlight the increased prevalence of asthma-related health crises within the pediatric population of the IE. Additionally, it is worthwhile to note that the hospitalization rates—across both the state and individual counties—are significantly more pronounced amidst the younger, 0–4 age group (Figure 2). Comparing the hospitalization rates across age groups, San Bernardino’s rate is 24.4% for the 0–4 age group (compared to 7.6% for the 5–17 age group), and Riverside’s rate is 14.4% for the 0–4 age group (compared to 6.8% age group).

The elevated prevalence of asthma hospitalizations among younger children may be attributed to several factors, such as underdeveloped immune systems, frequent misdiagnosis of asthma as respiratory tract infections, and narrower airways. Conversely, across the state and the four counties of SB, Riverside, Alameda, and Orange, asthma hospitalization rates are all notably decreased for the 18+ adult population (Table 4). Although both San Bernardino (SB) and Riverside (RS) counties are part of the IE, SB exhibits a higher burden of pediatric asthma both within the IE and general Southern California. Regardless of location, children aged 0–4 remain the most vulnerable, with SB also demonstrating the highest rates (Figure 2).

**Figure 3:**
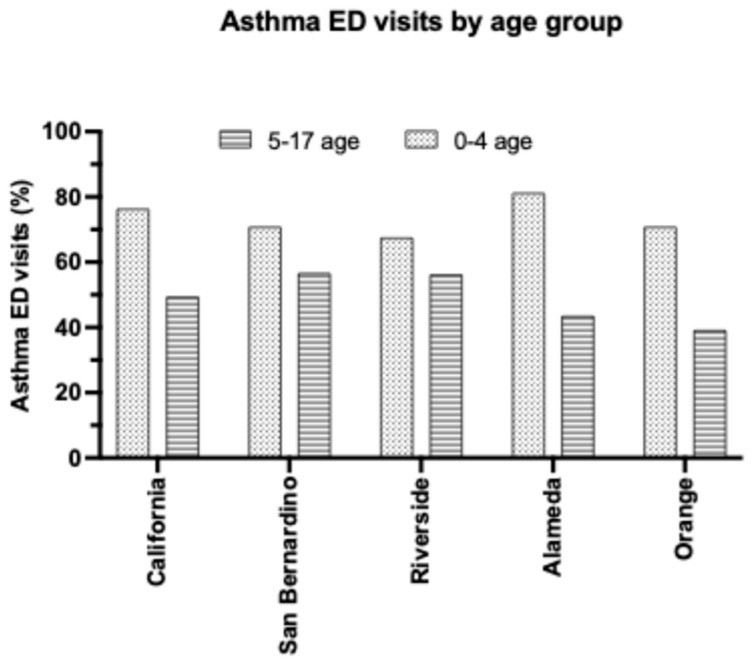
County–dependent variations in the rates of children ED visits due to asthma complications. Asthma-associated ED visits (in %) for the 0–4-year group vs. 5–17-year-old group shows an elevated trend of ED visits in the 5–17 yo group in IE. It also shows a lower trend of ED visits in the age group 0–4 in IE compared to the rest of the counties, which may be consistent with low access to health care (Source: California Department of Public Health).

Within the IE, region-specific symptoms for children presenting to the ED remain unreported in the literature. However, elevated environmental contributors in the IE may help explain the high asthma ED visit rates, and the clinical presentations in the IE are expected to mirror those in other regions. Asthma ED visit rates (categorized per primary discharge diagnosis codes) in 2022 in SB for children 0–17 years old was 60.5% and Riverside 59.3%, compared to the California statewide average of 56.7% (Table 5) [36]. Amidst the 0–4 age group, the statewide ED visit rate was notably 76.4% (compared to 49.5% for 5–17-year-olds), San Bernardino at 70.9% (56.7% for 5–17-year-olds), and Riverside at 67.6% (56.3% for 5–17-year-olds). There exists a similarity between asthma hospitalization and ED visit rates in that 1) younger children (i.e., ages 0–4 years old) are more likely to experience exacerbated asthma symptoms that require hospital care and 2) the SB tends to exhibit consistently elevated asthma ED and hospitalization rates compared to other counties in California (Figure 3). Although Riverside is part of the Inland Empire, its hospitalization rates are significantly lower than San Bernardino’s. As such, targeted prevention and resource strategies should prioritize San Bernardino. Overall, the IE is greatly affected, yet it is worthwhile to note that California as a state has an elevated asthma ED visit rate, potentially signaling a greater issue at hand.

Although asthma is a health concern for the pediatric population of California, children in the IE—especially SB—suffer the most. These findings highlight the significant burden of asthma in the Inland Empire, a region with high air pollution and limited access to health care. Asthma-related hospitalizations and ED visits in the IE are notably higher than the state average. The elevated asthma rates amidst school-aged children further emphasize the crucial need for interventions aimed at improving air quality, enhancing asthma management in schools, and increasing access to preventive health care. For instance, asthma severity and exacerbations impart a detrimental effect upon academic performance. In-depth analysis of the 2011–2014 California Health Interview Survey for children aged 5–11 underscored that of 715 respondents representing an estimated 314,200 California schoolchildren with asthma, approximately 50.3% students missed at least one school day, and 11.7% missed more than nine days of school due to asthma [37]. The combined effects of environmental pollutants and limited health care access in the region significantly contribute to worsened asthma outcomes, ultimately leading to increased school absences and compromised academic achievement for affected students. Moving forward, it is crucial to explore the specific causes behind these high asthma rates to develop tailored strategies that can reduce asthma-related health outcomes within the pediatric population of the IE.

## 4. Contributing Factors to Health Challenges in Pediatric Asthma in the Inland Empire

### 4.1. Environmental Exposure: IE as a Major Transportation Corridor

The IE is a major transportation hub within Southern California, identified by its proximity to major highways—including but not limited to Interstates 10, 15, 210, and 215—and freight railyards, such as the Colton and San Bernardino railyards. Earlier health and academic reports emphasized the high asthma rates and the consequent lack of adequate health care infrastructure, such as insurance and the availability of specialists [38]. A 2019 survey conducted by California State University, San Bernardino reported that the median cumulative commute time for IE residents was 48 min, with over half commuting more than an hour each day [39]. The increase in warehouse centers and the associated truck trafficking in the past 5 years have exacerbated the air pollution crisis in the IE [40]. These developments have sparked significant concerns about environmental impacts, traffic congestion, and quality of life. Specifically, more than 1 billion square feet of warehouse space was added in the IE as of 2023 [41]. These developments led to over 200 million diesel truck trips per year, producing over 300,000 pounds of diesel particulate matter, 30 million pounds of nitrogen oxide, and 15 billion pounds of carbon dioxide [41]. The greatest concern is derived from the statistic that 640 schools are situated within a half-mile radius of a warehouse in the South Coast Air Basin (SCAB, which includes Orange County, LA county, Riverside County, and SB) [41]. This has placed residents—especially children—at ever higher risks of asthma and cancer. These extensive changes increased vehicle emissions and regional air pollution, but also contributed to higher rates of asthma frequency, particularly among vulnerable pediatric populations. Therefore, another important aspect to accentuate is the role of geospatial proximity.

A 2015 examination of railyard proximity and adverse respiratory effects emphasized that children in the “exposure” school (i.e., located approximately 500 m from the San Bernardino Railyard) presented with a 33% higher likelihood of having an abnormal Fe_NO_ value in comparison to the “nonexposed” schoolchildren located 7 miles away [42]. For reference, the normal range of Fe_NO_ was suggested at 20 ppb. Fe_NO_ has previously been utilized as a screening marker for traffic-associated air pollution levels, with recent ongoing evidence bolstering its credibility as a sensitive measure for eosinophilic airway inflammation for the elevated risk and diagnosis of asthma [43]. In particular, increased levels of Fe_NO_ have been linked to new-onset asthma, even after adjustment for variables such as race, ethnicity, and family history of asthma [44]. In the Inland Empire, however, there is a notable absence of recent Fe_NO_ data from the past two years, underscoring a significant research gap in a region with high asthma prevalence. Moreover, children who lived at their current address for at least 6 months were associated with a 41% increase in low peak expiratory flow (an indicator of increased airway obstruction) and a 44% increase in airway inflammation [42]. This paints an alarming picture of how environmental exposures near major pollution sources contribute to early airway inflammation, increasing the risk of asthma development in vulnerable pediatric populations.

A 2018 study conducted by the Loma Linda School of Public Health examined residential proximity to 18 major California freight railyards—including the San Bernardino railyard—with respect to asthma-induced pediatric ED visits. Children living within the 0–5-mile radius of a railyard were discovered to be at significantly heightened risk for asthma-associated ER visits, with a notable percentage—approximately 40.8%—of the children pertaining to the 1–4-year age group [45]. Hence, the likelihood of a child visiting the ED for an asthma-related concern is strongly associated with residential proximity to a railyard. The high prevalence of asthma among younger children is especially concerning due to the long-term health risks it poses during a critical stage of growth and development. Furthermore, within the asthma-associated ED visits, an overwhelming 72.8% were of ethnic/racial minority background, of which 51.6% held Medicaid insurance [45]. This indicates a strong correlation between geographical vicinity and asthma-dependent ED visits, with household income as an underlying cofounding variable. However, it is necessary to note that this study includes several limitations. A central element is the amount of time children spend at their respective residences. For instance, some children may have been born at that address and lived there continuously, which could result in more severe asthma symptoms and frequent ED visits compared to a child whose family may have moved to the vicinity recently. Consequently, it is imperative to also quantitatively evaluate “in-home” factors that may contribute to exacerbated respiratory functions and the development of asthma, such as exposure to secondhand smoking.

### 4.2. Socioeconomic Factors

Socioeconomic factors, specifically with respect to household income, racial disparities, and parental education, exert a significant influence on the prevalence of asthma within the IE. On a broader scale, numerous academic studies have established that lower socioeconomic status (SES) is a strong predictor of morbidity and premature mortality globally [46]. A lower socioeconomic background can be considered an adverse determinant for preventative care and a risk for poor health. For children undergoing a critical period of growth (i.e., physical, social, and behavioral), socioeconomic disadvantage has been linked to a higher prevalence of adverse health outcomes. For instance, a study from Portugal’s Generation XXI birth cohort found that children from lower SES backgrounds exhibited increased rates of obesity, hypertension, and language/speech problems by the age of four [47]. These findings emphasize the significant role of socioeconomic factors in shaping health trajectories during critical periods of development. Specifically, asthma prevalence is elevated among low-income children, who also experience a more severe form of the disease. This increased severity is linked to greater health care utilization and higher associated medical costs [24].

Across 61 studies encompassing approximately 1,145,704 patients worldwide, lower SES was associated with increased asthma-induced ED visits, exacerbations, and mortality [48]. A 2010 UCLA study—and the most current detailed analysis—of income disparities and asthma care in California revealed that amongst Californians with asthma (equivalent to 4.9 million per 2007 CHIS data), approximately 46.6% were low-income children aged 1–11 and 38% low-income children aged 12–17 [49]. This is compared to just 31.3% of 18–64-year-olds [49]. These alarming figures emphasize a critical need for policy reform, resource allocation, and community-based interventions to protect low-income children, who remain at significantly higher risk for poor asthma outcomes without swift action.

In the IE, there is an overwhelming lack of research as to the precise extent of how low SES background has definitively contributed to the continuous rise of pediatric asthma. However, with increasing distance from railyards, even at 25–30 miles away, the percentage of Medicaid-insured children decreases to 39.3%, compared to 51.6% among those living within a 5-mile radius [45]. In California, Medicaid eligibility is determined by a household income less than at least 138% of the poverty level [50]. As of 2023, approximately 55.9% of children aged 0–18 in San Bernardino County were enrolled in Medi-Cal, California’s Medicaid program, while approximately 80–85% of California Children Services (CCS)-eligible children in San Bernardino County are also Medi-Cal-eligible (Table 6) [51].

Utilizing Medicaid as a marker of low SES, it becomes evident that lower SES is associated with greater exposure to environmental risk factors and ultimately worse asthma outcomes among children. This claim is strengthened by a 2013 finding that within the Southern California region—including the Riverside and San Bernardino counties—children from regions with lower SES were more susceptible to not only PM2.5 exposure, but also exhibited a higher likelihood of respiratory-associated hospitalizations, including asthma [52]. The association between lower SES and an escalated incidence of pediatric asthma can plausibly be explained by a gap in resources, such as a lack of reliable transportation and under-prescription of preventative inhalers. Furthermore, it is also worthwhile to note that this finding is not just limited to the Inland Empire. A retrospective study conducted in South Korea by Lee et al. showed that amongst 1,537,066 children from five different subtypes of SES, children of lower SES experienced more frequent asthma exacerbations, medical facility use, and admissions compared to their counterparts with higher SES [53].

Disparities in asthma prevalence across racial groups may further demonstrate the heightened burden of pediatric asthma within the IE. According to the CDC, the most recent asthma data collected in 2021 revealed that Hispanic children had an asthma mortality rate 1.4-times higher than that of non-Hispanic White children (Table 7) [54].

Narrowing the focus to the Inland Empire, per the 2020 US Census, the IE consists of more Latino/Hispanic residents than the rest of California, with 49.72% in Riverside County, 55.9% in San Bernardino County, and 40.4% statewide [55]. The IE is also home to a significant immigrant population, and as of 2023, approximately 21.4% (468,000 people) of people in the San Bernardino County are foreign-born [55]. For children born into these families, the heightened occurrence of asthma could be attributed to a variety of factors, such as language barriers hindering communication, lack of insurance, and limited availability of culturally competent care. Javier et al highlighted that children in immigrant families were more likely to lack a usual source of care, report delays in medical care, and report no visit to the doctor compared to their non-immigrant counterparts [56]. These factors contribute to delayed treatment and increased reliance on emergency services.

### 4.3. Limitations of Parental Education

Low parental health literacy is a significant determinant of the elevated prevalence of pediatric asthma and increased utilization of ED services. Limited health literacy amidst parents impairs their ability to understand asthma management protocols, effectively recognize early symptoms, and administer preventive therapies effectively, thereby contributing to suboptimal disease control in children. Dewalt et al. reported that children of parents with low literacy exhibited higher rates of moderate to severe persistent asthma, as well as increased ED visits and hospitalizations, independent of confounding socioeconomic factors [57]. Within the IE, educational attainment is markedly lower than the state and national averages. Only 22.9% of adults hold a bachelor’s degree, with Latino adults demonstrating the lowest rates of higher education achievement, with 11.7% holding a bachelor’s degree and 3.3% graduate degrees [58]. These educational disparities are strongly correlated with reduced health literacy, further impeding pediatric asthma management. Addressing parental health literacy deficits through culturally tailored interventions remains a critical strategy for mitigating asthma-related health disparities among children in the IE.

### 4.4. Limitations in the Current Health Care Infrastructure

Despite the region’s ever-growing population, the IE’s health care infrastructure presents significant challenges in effectively managing pediatric asthma, due to a shortage of pediatric specialists, limited academic medical centers, and insufficient community-based asthma programs [38]. The IE has the second-lowest supply of specialists in the state at 83 specialists per 100,000 residents (compared to the statewide average of 131 per 100,000), which significantly impacts access to timely and appropriate care for pediatric asthma patients [59]. This shortage is further exacerbated by the region’s rapid population growth. This scarcity can also be attributed to the limited presence of academic medical centers and children’s hospitals, which are critical for providing comprehensive care and facilitating treatment. For instance, Loma Linda University Children’s Hospital, which is the only dedicated children’s hospital for more than 1.2 million children in the IE [60], offers a network of pediatric specialists, including those in allergy, immunology, and pulmonology. As a result, children with asthma often experience delays in receiving specialized care, leading to increased ED visits and hospitalizations.

Furthermore, the IE lacks robust community asthma programs and educational initiatives for preventive care and patient education [38]. Various efforts, like the Riverside County Childhood Asthma Program and the Family Asthma Program by the Inland Empire Health Plan, strive to provide individualized asthma education. However, these initiatives are not substantial enough to address the needs of the entire pediatric population. One potential solution is a medical–social coordination model that has care coordinators providing asthma education, connecting families to health care and social support services, and enhancing communication between patients and clinicians. In fact, implementation of this type of coordination model resulted in a 2.2 symptom day reduction for the intervention group, alongside significant decreases in daytime and night-time symptom days over 2 weeks of 45.1% and 46%, respectively [61].

Implementing a similar approach in the Inland Empire has the potential to significantly reduce asthma-related exacerbations, hospitalizations, and school absenteeism. Adjusting these programs to address the region’s unique demographic challenges (i.e., high rates of poverty, immigrant populations, and limited access to specialty care) would be necessary for maximizing effectiveness and improving long-term health outcomes for pediatric asthma patients in the IE.

Comprehensive community-based asthma interventions that incorporate multiple components, such as self-management education, home environmental assessments, and coordinated care, have been associated with improved asthma-related outcomes. Specifically, these programs are linked to reductions in ED utilization, hospital admission rates, and frequency of symptomatic days. Addressing these infrastructural limitations requires strategic investments in health care resources, including but not limited to the expansion of pediatric specialty services, the establishment of additional pediatric-dedicated medical centers, and the development of comprehensive community-based asthma programs. For example, beginning in 2022, Medi-Cal expanded its asthma treatment services to include unconventional” at-home treatments, such as installation of air purifiers in homes, removal of mold, and replacing old carpeting [60]. These interventions aim to reduce environmental triggers that worsen asthma symptoms, offering a more holistic, family-friendly approach to management.

## 5. Gaps in the Literature

While substantial progress has been made over the years in understanding the epidemiology and management of pediatric asthma, several gaps remain, especially regarding underserved and environmentally burdened populations residing in the IE. Asthma prevalence has been extensively studied in urban centers such as Los Angeles. However, there exists an under-representation of research in the IE, which faces a unique combination of socioeconomic, health care, and environmental challenges. The lack of region-specific data severely hinders the development of targeted interventions that are tailored to the needs of the community.

Although environmental exposures such as PM2.5 and ozone have been linked to asthma exacerbations, few studies have provided a comprehensive examination of the long-term effects of chronic exposure in pediatric populations living in areas with persistent poor air quality, such as the IE. Existing studies are more focused on short-term health outcomes, leaving a gap in understanding the cumulative effect of lifelong exposure. Research has also been limited regarding indoor exposure to environmental factors, such as mold and poor housing conditions, which involves pediatric asthma populations from economically disadvantaged backgrounds that are prevalent in the IE, where housing is suboptimal.

There is also a lack of nuanced studies investigating the role of geographic proximity to industrial sites, highways, and railyards in shaping pediatric asthma outcomes. Previous studies have demonstrated that children living near such pollution sources are at a heightened risk for asthma attacks and hospitalizations. There is a need for more precise data that examines how various environmental exposures interact with socioeconomic factors and can be translated into effective public health strategies.

While the relationship between access to care and asthma outcomes has been well established, there is a lack of studies exploring specific barriers faced by pediatric asthma populations in the IE. These barriers are also likely compounded by the region’s low-income status and high percentage of uninsured and underinsured residents. The intersection of race and health care access in the IE also remains unexplored, given the area’s high prevalence of Hispanic and African American populations. These factors likely contribute to the cycle of delayed diagnosis, management, and asthma control in these communities.

The lack of longitudinal and region-specific data on pediatric asthma further exacerbates the challenge of developing targeted public health interventions. Most studies on pediatric asthma in California emphasize broader state and national trends. Yet, there exists a pervasive lack of emphasis upon localized dynamics of areas such as the IE. This gap in the literature limits the effectiveness of asthma intervention programs and public health policies to better address the unique needs of high-risk areas.

To address these gaps, future research should emphasize longitudinal studies that focus upon the health outcomes of children exposed to the specific environmental risks present in the IE, particularly in terms of both outdoor and indoor pollutants. Another area of investigation could be the effect of asthma on other critical aspects of growth, such as the auditory and nervous systems. The synergistic effects of environmental, socioeconomic, and health care-related factors on asthma prevalence and severity should be considered. Addressing regional disparities in health care access through community-based interventions and policy advocacy will be essential in improving pediatric asthma outcomes in the IE. Firstly, one consideration is stricter emissions control near schools, railyards, and major transportation zones in the IE that could substantially reduce pediatric exposure to particulate matter and ozone. Second, investment in community-based asthma surveillance systems tailored to the needs of the IE would provide real-time data to identify high-risk neighborhoods and evaluate the impact of interventions. Third, the expansion of school-based asthma programs that provide education, preventative inhaler use, and home environmental assessments would help bridge current care gaps through both children and family-centered efforts. Ultimately, policy reforms to improve health care access, including funding for additional pediatric specialists, expanded Medi-Cal coverage for preventative services, and integration of social determinants screening into pediatric visits, are all critical for addressing systemic inequities and the root causes of disparities.

## 6. Future Directions

Future research should prioritize investigation of region-specific elements, such as the seasonal Santa Ana winds, to develop tailored clinical strategies that will improve long-term outcomes for vulnerable pediatric patients in the IE. Beyond region-specific research, these findings underscore the urgent need for multilevel interventions. Policy measures such as emissions reduction near sensitive land surrounding schools and childcare centers, stricter zoning regulations for warehouses and freight corridors, and dedicated funding for pediatric asthma registries in the IE could meaningfully reduce disease burden. From a clinical standpoint, expanding community health worker programs, school-based asthma management initiatives, and telehealth-based specialty access may provide feasible pathways to improving care access in resource-limited regions. These recommendations, alongside continued surveillance efforts and longitudinal research, represent actionable next steps toward reducing the disproportionate asthma burden among children in the IE.

## 7. Conclusions

Children are especially susceptible to environmental pollution due to their developing systems (e.g., brain and cognitive skills), higher respiratory rates (i.e., increased pollutant intake), and less developed biological defenses (i.e., immature detoxification and immune systems). Moreover, children possess minimal capacity to regulate or avoid exposure to these environmental hazards.

While pediatric asthma remains the most common chronic disease worldwide, within the IE, pediatric asthma is a pressing matter of concern since it significantly impairs the normal development of children. In addition, pediatric asthma places a significant burden on the health care system, exerts a long-lasting impact on adolescent learning and achievement, and affects underserved communities. Here, we highlight the unique determinants underlying the high prevalence of asthma in the 0–17-year-old population within the IE/SB, which includes but are not limited to heightened environmental risks (i.e., indoor factors such as allergies and molds as well as pollutants measured per PM2.5), socioeconomic barriers, and lack of targeted public health initiatives and specialist-oriented care. It is well established that there continues to be a higher-than-average prevalence of pediatric asthma in the IE/SB. We also pointed out the need for more data collection and research to better help improve the public health determinant of the IE population.

## Figures and Tables

**Table 1:** Key environmental pollutants linked to increased risk of developing pediatric asthma [6].

Pollutant	Source(s)	Health Effects in Children
Particulate Matter (PM2.5)	Vehicle emissions, industrial activities, wildfires	Triggers asthma attacks; impairs lung development; increases ER visits and hospitalizations
Ozone (O_3_)	Photochemical reactions from vehicle exhausts and sunlight	Causes airway inflammation; exacerbates asthma symptoms; reduces lung function
Nitrogen Dioxide (NO_2_)	Traffic emissions, industrial combustion	Increases asthma incidence; worsens symptoms; linked to chronic respiratory issues
Sulfur Dioxide (SO_2_)	Industrial processes, fossil fuel combustion	Triggers bronchoconstriction; increases asthma-related ER visits
Carbon Monoxide (CO)	Incomplete combustion of fossil fuels	Reduces oxygen delivery; aggravates respiratory conditions
Volatile Organic Compounds (VOCs)	Paints, cleaning products, gasoline vapors, vehicle exhaust	Contribute to ozone formation; linked to respiratory irritation and asthma exacerbation

**Table 2:** Key indoor pollutants increasing pediatric asthma risk [15].

Pollutant	Source(s)	Health Effects in Children
Pest Allergens	Cockroaches, rodents in substandard housing	Linked to increased asthma severity and frequency of attacks
Mold Spores	Damp or poorly ventilated conditions	Triggers allergic reactions to asthma exacerbations
Endotoxin	Bacterial byproducts in dust from poor sanitation	Cause airway inflammation and contribute to chronic asthma symptoms

**Table 3:** Most Polluted Cities in North America. The top 5 most polluted cities in Northern America as per the 2024 World Air Quality Report. Apart from Huntington Park, all the cities are all located in the Inland Empire.

Rank	City	County	PM2.5 (µg/m^3^) *
1	Ontario, CA	IE	14.3
2	Bloomington, CA	IE	13.9
3	Huntington Park, CA	LA	13.6
4	San Bernardino, CA	IE	12.9
5	Fontana, CA	IE	12.7

* Except for Huntington Park, all the cities are all located in the IE (Source: IQ Air 2024 World Air Quality Report).

**Table 4:** Asthma Hospitalization Rates in Four Counties of California in 2022. The table shows the rates of asthma hospitalization (per 10,000 residents) across the four age groups (in years): 0–4, 0–17, 5–17, and ≥18. The data was based on California Breathing—the state asthma program run by the California Department of Health Care Access and Information.

County	Asthma Hospitalization Rate for ≥18 Population	Asthma Hospitalization Rate Within the 0–17 Age Group	Asthma Hospitalization Rate Within the 0–4 Age Group	Asthma Hospitalization Rate Within the 5–17 Age Group
California	2	9	17.3	5.9
San Bernardino (IE)	2.4	9.6	24.4	7.6
Riverside (IE)	1.8	8.8	14.4	6.8
Alameda (NorCal)	1.7	7.1	16.4	3.6
Orange (SoCal)	1.2	7	14.6	4.2

Source: California Breathing—the state asthma program run by the California Department of Health Care Access and Information.

**Table 5:** County-dependent variations in the rates of pediatric ED visits due to asthma complications. Asthma-associated ED visits (in % per 10,000 residents) for the 0–4 group vs. 5–17 group show an elevated trend of ED visits in the 5–17 group in IE. They also show a lower trend of ED visits in the age group 0–4 in IE compared to the rest of the counties, which may be consistent with low access to health care. The highlighted part captures elevated ED visit rates in SB across all age groups. (Source: California Department of Public Health).

County	Asthma ED Visit Rates for 18+ Population	Asthma ED Visit Rates for 0–17 Age Group	Asthma ED Visit Rates Within the 0–4 Age Group	Asthma ED Visit Rates Within the 5–17 Age Group
California	25.1	56.7	76.4	49.5
San Bernardino (IE)	30.9	60.5	70.9	56.7
Riverside (IE)	26.5	59.3	67.6	56.3
Alameda (NorCal)	25.5	53.7	81.3	43.6
Orange (SoCal)	14.3	47.7	70.9	39.2

**Table 6:** Medi-Cal Enrollment Among Children in San Bernardino County. (Source: San Bernardino County—Department of Public Health, 2023).

Population Group	Percentage Enrolled in Medi-Cal	Notes
All children (ages 0–18)	55.90%	Enrolled in Medi-Cal as of 2023
CCS-eligible children	80–85%	Also eligible for Medi-Cal

**Table 7:** Population-dependent Variations in Pediatric Asthma Related Mortality. Asthma Mortality rate in children (age < 18 years old) in 2021 by race/ethnicity further demonstrate the heightened burden of pediatric asthma within the IE. (Source: Centers for Disease Control and Prevention).

Race/Ethnicity	Death Rate Due to Asthma Per Million (SE) ^1^
White—Non-Hispanic	1.0 (0.17)
Black—non-Hispanic	7.7 (0.87)
American Indian or Alaska Native—Non-Hispanic	N/A ^2^
Asian, Native Hawaiian, Pacific Islander—Non-Hispanic	N/A ^2^
Hispanic	1.4 (0.27)

^1^: Asthma as the cause of death (ICD-10 codes J45–46). ^2^: Data has been suppressed when the number of deaths is 9 or fewer.

## Data Availability

Not applicable.
